# Adaptability and stability of Coffea canephora to dynamic environments using the Bayesian approach

**DOI:** 10.1038/s41598-022-15190-x

**Published:** 2022-07-08

**Authors:** Fabio Luiz Partelli, Flavia Alves da Silva, André Monzoli Covre, Gleison Oliosi, Caio Cezar Guedes Correa, Alexandre Pio Viana

**Affiliations:** 1 Centro Universitário Norte do Espírito Santo, Universidade Federal do Espírito Santo, 29932-540 São Mateus, Espírito Santo, Brasil; 2 Laboratório de Melhoramento Genético Vegetal, Universidade Estadual do Norte Fluminense Darcy Ribeiro, 28013-602 Campos dos Goytacazes, Rio de Janeiro, Brasil; 3 Centro de Ciências Agrárias Engenharias, Universidade Federal do Espíerito Santo, 29500-000 Alegre, Espírito Santo, Brasil

**Keywords:** Computational biology and bioinformatics, Plant sciences

## Abstract

The objective of this work was to use the Bayesian approach, modeling the interaction of coffee genotypes with the environment, using a bisegmented regression to identify stable and adapted genotypes. A group of 43 promising genotypes of Coffea canephora was chosen. The genotypes were arranged in a randomized block design with three replications of seven plants each. The experimental plot was harvested four years in the study period, according to the maturation cycle of each genotype. The proposed Bayesian methodology was implemented in the free program R using rstanarm and coda packages. It was possible to use previous information on coffee genotypes as prior information on parameter distributions of an Adaptability and Stability model, which allowed obtaining shorter credibility intervals and good evidence of low bias in the model by the determination coefficient. After fine adjustments in the approach, it was possible to make inferences about the significant GxE interaction and to discriminate the coffee genotypes regarding production, adaptability, and stability. This is still a new approach for perennials, and since it allows more accurate estimates it can be advantageous when planning breeding programs. The Z21 genotype is recommended to compose part of selected genetic material for highly technical farmers, as it responds very well to the favorable environment, being one of the most productive and with excellent stability.

## Introduction

Worldwide, around 174 million bags of coffee are produced every year. Of this total output, 59.8% is Arabica (Coffea arabica) and 40.2% Robusta/Conilon (*Coffea canephora*)^1^. The entire coffee chain accounts for around 172,000 million U$D annual revenue, and Brazil for approximately 32% of the global production^2^.

Conilon coffee is commonly vegetatively propagated, which warrants a uniform crop development and better fruit quality, with high genetic variability between cultivars. These genotypes differ in their interaction with the environment, making breeding a challenge^3–5^.

The ultimate goal of plant breeding programs is to recommend cultivars for commercial use. To do it is necessary to model the behavior of genotypes across environments, to get greater confidence and better based-decision about the recommendations of superior cultivars. This modeling of Genotype x Environment interaction (GE) can be defined as the differential response of genotypes to environmental variation. It is necessary to discriminate cultivars as to the most stable ones, that is, those with less significant GE, or those more adapted to specific environments as more responsive to a good environment^6^.

The identification and quantification of the factors that affect GE are key elements for the understanding of phenotypic adaptation. In breeding programs, these factors are important because there is always a need to select genotypes in multi-environmental trials. Analytical methods linked to multi-environmental characterization protocols allow useful inferences to decision making, such as the identification of environmental factors that determine the GE interaction, and mainly the adaptability and stability of genotypes. The objective is to identify individuals with predictable behavior and who are responsive to environmental variations, under specific or broad conditions^7–9^.

There are currently several methodologies available in studies of adaptability and stability, varying in terms of the concepts employed and statistical principles. All are based on the existence of the GE interaction and the choice depends on the experimental data, the number of available environments, required precision, and the type of information desired^10^.

The simplest method is based on splitting the sum of squares of the effects of environments from a joint analysis of the effects of environments within each genotype. Other methods may use some abstractions other than this, such as the arithmetic mean of the variance components^11^, and decomposition of the sum of squares in isolated genotypes^12^. Later, methods based on linear regression were proposed^13,14^, where for each genotype the dependent variable regresses according to an environmental index defined as the average of all genotypes in the environment. Subsequently, a correction was proposed due to the errors implicit in the measurements of the genotypes themselves, where the independent variable was associated with errors, invalidating the estimator^15^. Subsequently, it was proposed to evaluate the genotypes in favorable and unfavorable environments to identify the ideotype, using a regression pair^16^.

With this approach, there is a principle that the ideotype would be the one with high production capacity, high stability, little sensitivity to the adverse conditions of unfavorable environments, but capable of satisfactorily responding to the improvement of the environment. Taking a single slope as in the conventional methodology, this ideotype would be discarded if all environments were considered. On the contrary, if the slope is double, deviations from different environments can be estimated, decreasing the error about an estimated mean line. However, this method is impractical when there are a small number of environments in the subgroups. It was then proposed to use again a single regression, but bisegmented^17^. This method was later extended to a simpler operation and with statistical properties more suited to the purpose of improvement^18^.

More recently, other methods have also been proposed as Additive Multiplicative Models Interaction – AMMI^19^, GGE Biplot^20^, Extended Centroid Method^21,22^, and non-parametric analysis^23^. Although there are many methods available, these procedures have limitations in dealing with unbalanced data, non-orthogonal experiments (incomplete blocks), and heterogeneity of variance between various environments.

Some new approaches help to circumvent these problems, including Intelligence Artificial^24,25^ and based on Bayesian approaches^26–28^. These approaches are particularly useful to avoid false positives when few environments (n < 10) are evaluated. Both approaches can be combined with other techniques (regression), but the Bayesian approach allows for the incorporation of additional information about the parameters through prior distributions incorporated into a model with a probability distribution. In practice, this information can be obtained using information from previous studies^29^, improving the model goodness of fitting^30^. Plant breeders can now gain new insights by leveraging previously published studies, and also leveraging information from past experiments within programs. Other reviews may provide more relevant information on the use of the Bayesian approach^31,32^.

In coffee plants, as well as other perennial fruit trees worldwide appreciated, situations such as those described above are commonly encountered, such as unbalanced, non-orthogonal data and few data available. Allied to this, is the time needed to obtain data on perennial plants, which makes it very difficult to carry out large experiments in different locations. So, we use an approach not yet reported for coffee. The objective was to use the Bayesian approach by modeling the interaction of coffee genotypes with the environment using a bisegmented regression to identify stable and adapted genotypes. We also provide a workflow for implementation in free and open-source R software and prior estimates for breeders worldwide.

## Methodology

### Plant material

A group of 43 promising *C. canephora* genotypes was chosen, most of which had been selected by coffee farmers in the State of Espírito Santo – Brazil (Supplementary Table S1). These 42 cutting and one seed-propagated genotype were propagated again by cuttings and planted in an experimental plot for selection for high yield potential and agronomic traits of interest. The experiment was planted in April 2014, at a spacing of 3.5 m × 1.0 m, totaling 2,857 plants^.^ha^−1^, in the county of Itabela, Bahiaype, Brazil (lat 16° 36′ 52.00″ S, long 39° 30′ 33.00″ W, alt 140 m asl). According to Köppen’s classification, the regional climate is Aw tropical, with hot humid summers and dry winters^33^, mean minimum temperatures of over 15 °C (July and/or August), and mean maxima of over 35 °C in January and/or February in some years (Supplementary Fig. S1).

The genotypes were arranged in a randomized block design with three replications of seven plants each. The number of orthotropic branches was controlled by pruning, to maintain around 12,000—15,000 stalks per hectare. In all experimental years, mechanical and chemical weeding was performed at least once and insecticides and fungicides were applied. The experimental area was drip-irrigated in all evaluation years. Fertilizers (N, P_2_O_5,_ and K_2_O) were applied according to the plant requirements and phenological stages (600, 100, and 400 kg^.^ha^−1.^year^−1^, respectively).

The experimental plot was harvested four times in the study period (2016, 2017, 2018, and 2019), according to the maturation cycle of each genotype. The plots were harvested separately for each genotype, measuring the production in liters per plot. Later, the individual yield of each genotype was converted into bags of green coffee per hectare, based on two harvests and extrapolated to the others, based on the plant spacing yield per hectare was calculated.

### Statistical analyses

The data were subjected to analysis of variance in each environment, and the homogeneity of residual variance was later verified using Hartley’s test. Posteriorly was performed a joint variance analysis, by model:

1 $$ Y_{ijk} = { }\mu + {\text{Z }}\left( {r/e_{k\left( j \right)} } \right) + {\text{W}}\left( {e_{j} } \right) + {\text{Q}}\left( {g_{i} } \right) + {\text{T}}\left( {ge_{ij} } \right) + \varepsilon_{ijk} $$

where $Y_{ijk}$ is the vector of phenotypic observations of the $i^{th}$ genotype in the kth repetition in the $j^{th}$ environment; $\mu$ is the general mean; $r/e_{k\left( j \right)}$ is the vector of fixed effect of the kth repetition inside the $j^{th}$ environment, associated with the ${\text{Z}}$ incidence matrix and with the probability distribution $r/e\sim N\left( {\mu_{k\left( j \right)} ,\sigma_{r/e}^{2} } \right)$; $g_{i}$ is the vector of fixed effect of the $i^{th}$ genotype, associated with the ${\text{Q}}$ incidence matrix and with the probability distribution $g \sim N\left( {\mu_{i} ,\sigma_{g}^{2} } \right)$; $e_{j}$ is the vector of fixed effect of the ${j}^{th}$ environment, associated with the $\mathrm{W}$ incidence matrix and with the probability distribution $e \sim N({\mu }_{j},{\sigma }_{e}^{2})$; ${ge}_{ij}$ is the vector of fixed effect of the interaction of the ${i}^{th}$ genotype inside the ${j}^{th}$ environment, associated with the $\mathrm{T}$ incidence matrix and with the probability distribution $ge \sim N({\mu }_{ij},{\sigma }_{ge}^{2})$; and ${\varepsilon }_{ijk}$ is error not caught by model $\varepsilon \sim N(0,{\sigma }_{\varepsilon }^{2})$. Once a significant interaction between the effects of genotypes and environment was observed, a second modeling was carried out seeking the responses of the genotypes in favorable and unfavorable environments.

### Bayesian inference

The bisegmented regression model used is:

2 $$ Y_{ij} = { }\beta_{i0} + \beta_{i1} I_{j} + \beta_{i2} T\left( {I_{j} } \right) + e_{ij} $$

where $Y_{ij}$ is the response of genotype $i$ in environment $j$, $\beta_{i0}$ is the mean response of genotype $i$, $\beta_{i1}$ is the slope under the first regime (the linear regression coefficient related to the unfavorable environments), and $\beta_{i2}$ represents the change in slope from the first to the second regime ($\beta_{i1}$ + $\beta_{i2}$ is the slope after the change-point, that is, the linear response to the favorable environments). Further, $I_{j}$ is the coded environmental index, $T\left( {I_{j} } \right) = 0$ if $I_{j} > 0$, or $T\left( {I_{j} } \right) = { }I_{j} - \overline{I}_{ + }$ if $I_{j} > 0{ }$ and $\overline{I}_{ + }$ is mean of the coded environmental index considering only environments with positive indexes and ${e}_{ij}$ is the error term, NID $(0, {\sigma }^{2})$.

The modified bisegmented regression model^34^ showed can be performed by the Bayesian approach^6,28^, where the likelihood function and the joint posterior distribution can be founded. In summary, assuming $e_{ij} |I\sigma_{ie}^{2} \sim N\left( {0,I\sigma_{ie}^{2} } \right)$, each observation $y_{ij}$ has a distribution $y_{ij} \sim N\left( {\beta_{i0} + \beta_{i1} I_{j} + \beta_{i2} T\left( {I_{j} } \right);I\sigma_{ie}^{2} } \right)$, and the likelihood function for each genotype is given by:

3 $$ L_{i} \left( {\beta_{i0} ,\beta_{i1} ,\beta_{i2} ,\sigma_{ie}^{2} ,y_{ij} } \right) = \frac{1}{{\left( {\sqrt {2\pi \sigma_{ie}^{2} } } \right)^{a} }}exp\left\{ { - \frac{1}{{2\sigma_{ie}^{2} }}\mathop \sum \limits_{{I_{{\left\{ {I_{j \le 0} } \right\}}} }} \left[ {y_{ij} - \beta_{i0} - \beta_{i1} I_{j} } \right]^{2} - \frac{1}{{2\sigma_{ie}^{2} }}\mathop \sum \limits_{{I_{{\left\{ {I_{j > 0} } \right\}}} }} \left[ {y_{ij} - \beta_{i0} - \beta_{i1} I_{j} - \beta_{i2} \left( {I_{j} - I_{ + }^{ - } } \right)} \right]^{2} } \right\} $$

The prior distributions for the parameters $\left( {\beta_{i0} ,\beta_{i1} ,\beta_{i2} ,\sigma_{ie}^{2} } \right)$ are given by:

4 $$ \beta_{i0} |\mu_{{\beta_{i0} }} ,\sigma_{{\beta_{i0} }}^{2} \sim N\left( {\mu_{{\beta_{i0} }} ,\sigma_{{\beta_{i0} }}^{2} } \right) $$

5 $$ \beta_{i1} |\mu_{{\beta_{i1} }} ,\sigma_{{\beta_{i1} }}^{2} \sim N\left( {\mu_{{\beta_{i1} }} ,\sigma_{{\beta_{i1} }}^{2} } \right) $$

6 $$ \beta_{i2} |\mu_{{\beta_{i2} }} ,\sigma_{{\beta_{i2} }}^{2} \sim N\left( {\mu_{{\beta_{i2} }} ,\sigma_{{\beta_{i2} }}^{2} } \right) $$

7 $$ \frac{1}{{\sigma_{ie}^{2} }} = \tau_{ie} |\alpha_{1} ,\beta_{1} \sim Gamma\left( {\alpha_{i} ,\beta_{i} } \right) $$

where ${\mu }_{i0}{i0}^{i0},{\sigma }_{{\beta }_{i0}}^{2},{\mu }_{i1}{il}^{il},{\sigma }_{{\beta }_{il}}^{2},{\mu }_{i2}{i2}^{i2},{\sigma }_{{\beta }_{i2}}^{2}$, and ${\alpha }_{i},{\beta }_{i}$ are the known parameters. This last prior distribution is the Gamma distribution with mean and variance equal to $\frac{\alpha }{\beta }$ e $\frac{\alpha }{{\beta }^{2}}$, respectively. Additionally,${\sigma }_{ie}^{2}$ the precision is equal to $\frac{1}{{\sigma }_{ie}^{2}}$.

The joint posterior distribution is proportional to the product of the likelihood function (Eq. 3) and the prior distributions (Eqs. 4–7).

8 $$P\left({\beta }_{i0},{\beta }_{i1},{\beta }_{i2},{\tau }_{ie}=\frac{1}{{\sigma }_{ie}^{2}}|{y}_{ij}\right)\propto \frac{1}{{\left(\sqrt{{2\pi \sigma }_{ie}^{2}}\right)}^{a}}exp\left\{-\frac{1}{{2\sigma }_{ie}^{2}}\sum_{{I}_{\left\{{I}_{j\le 0}\right\}}}{\left[{y}_{ij}-{\beta }_{i0}-{\beta }_{il}{I}_{j}\right]}^{2}-\frac{1}{{2\sigma }_{ie}^{2}}\sum_{{I}_{\left\{{I}_{j>0}\right\}}}{\left[{y}_{ij}-{\beta }_{i0}-{\beta }_{i1}{I}_{j}-{\beta }_{i2}\left({I}_{j}-{I}_{+}^{-}\right)\right]}^{2}\right\} -\frac{1}{\sqrt{{2\pi \sigma }_{{\beta }_{i0}}^{2}}}exp\left[-\frac{1}{{2\sigma }_{{\beta }_{i0}}^{2}}{\left({\beta }_{i0}-{\mu }_{{\beta }_{i0}}\right)}^{2}\right]*\frac{1}{\sqrt{{2\pi \sigma }_{{\beta }_{i1}}^{2}}}exp\left[-\frac{1}{{2\sigma }_{{\beta }_{i1}}^{2}}{\left({\beta }_{i1}-{\mu }_{{\beta }_{i1}}\right)}^{2}\right]*\frac{1}{\sqrt{{2\pi \sigma }_{{\beta }_{i2}}^{2}}}exp\left[-\frac{1}{{2\sigma }_{{\beta }_{i2}}^{2}}{\left({\beta }_{i2}-{\mu }_{{\beta }_{i2}}\right)}^{2}\right]*\frac{{\beta }_{i}^{\alpha 1}{\tau }_{ie}^{{\alpha }_{1}-1}{e}^{{\beta }_{i}{\tau }_{ei}}}{\Gamma \left({\alpha }_{1}\right)}$$

To make inferences regarding the parameters in Eq. 8, the Markov chain Monte Carlo (MCMC) was used to obtain the posterior marginal distributions for each parameter. The marginal distribution samples of the stability parameter, $\sigma_{di}^{2}$, were obtained indirectly. This parameter is a function of $\sigma_{ie}^{2}$ according to the following expression: $\hat{\sigma }_{di}^{2} = \hat{\sigma }_{ie}^{2} - \frac{MSR}{r}$, where MSR is the residual mean square obtained from the variance analysis and r is the number of repetitions of the experiment.

We adopted four MCMC chains considering 100,000 iterations (nitt) each of the Gibbs sampler algorithm, after carrying out some convergence tests of the chains with a visual inspection of the behavior of the parameter estimates, starting at 10,000 iterations and increasing until stability is obtained. We set the burn-in to 10,000 iterations and thinned every four iterations (thin = 1:4). In each chain, we analyzed the posteriori mean, standard deviation, 95% credibility intervals, and convergence criterion statistics according to^35,36^. The methodology was implemented in software R^37^, and the joint distribution samples were obtained using the rstanarm::stan_glm package::function^38^, The MCMC chain convergence at 100,000 iterations was accessed by Geweke's criterion, according to the recommendations of^39^, using the coda::gewekediag package::function^40^.

### Prior’s distributions

We left from a minimally informative prior distribution as tested in the works described in the literature^41^, which were represented by distributions with large variances:

9 $$ \beta_{i0} |\mu_{{\beta_{i0} }} ,\sigma_{{\beta_{i0} }}^{2} \sim N\left( {\mu_{{\beta_{i0} }} = 0,\sigma_{{\sigma_{i0} }}^{2} = 100000} \right) $$

10 $$ \beta_{i1} |\mu_{{\beta_{i1} }} ,\sigma_{{\beta_{i1} }}^{2} \sim N\left( {\mu_{{\beta_{i1} }} = 0,\sigma_{{\sigma_{i1} }}^{2} = 100000} \right) $$

11 $$ \beta_{i2} |\mu_{{\beta_{i2} }} ,\sigma_{{\beta_{i2} }}^{2} \sim N\left( {\mu_{{\beta_{i2} }} = 0,\sigma_{{\sigma_{i2} }}^{2} = 100000} \right) $$

12 $$ \tau_{ie} |\alpha_{i} ,\beta_{i} \sim Gamma\left( {\alpha_{i} = 0.001,\beta_{i} = 0.001} \right) $$

Later we also tested another approach used in the literature^28,42–44^, which was characterized by the estimates obtained from the frequentist analysis of the bi-segmented model, used as information to define the hyperparameters. But we take advantage of the biggest advantage of the Bayesian approach, which is to be able to incorporate information a priori from previous data, and we use the data described in our previous works^4,45–47^ to make inferences about hyperparameters in distributions.

The model (hyperparameters) was chosen considering the one that presented more accurate metrics about the chosen prior. The prior choice was justified as suggested by^48,49^, with the modification of the alpha.mu and alpha. Vex tension parameters for variances and values to means, based on the lowest Deviance Information Criterion (DIC) value among the models, where $DIC = D\left( {\hat{\theta }} \right) - 2p_{D}$. Here, $D\left( {\hat{\theta }} \right)$ is a point estimate of the deviance obtained by replacing the parameters with their posterior mean estimates in the likelihood function and $2p_{D}$ is given by the effective number of parameters in the models. A lack of increment in the informative capacity of the prior was considered when |Δ|< 2 between the DIC of the models^50^. To help the a priori decision making, the predictive capacity of the model was also considered, in cross-validation, with ten folds (90% of training, and 10% of validation). Thus, the average correlation between the response of genotype $i$ in environment $j$ and phenotypic average observed from the cross-validation folds was considered.

## Results and discussion

When evaluated in an environment, the individual phenotypic value is the result of the action of the genotypic effect under the influence of the environment to which it is submitted. However, when evaluating the same individual in different environments, an additional component often appears that influences its phenotypic value, which is called the interaction between genotypic and environmental effects (GE). This interaction quantifies the differentiated behavior of the genotypes in the face of environmental variations. The GE interaction is one of the biggest problems of breeding programs of any kind, both in the selection and selection phase and in the genotype recommendation stage. In the coffee genotypes evaluated in this work, it was possible to observe that there was a significant GE interaction (P < 0.001) for the production of bags of green beans per hectare (Table 1).

**Table 1:** Mean squares from joined analysis of variance for the yield of 43 coffee genotypes assessed in four environments. Harvest 2016, 2017, 2018, and 2019.

Factor	DF	MS	F value	Pr(> F)
Environment (E)	3	153,585	267.216	2E-16***
Repetition (R)	2	546	0.95	0.388
Genotypes (G)	42	6674	11.612	2E-16***
R/E	6	790	1.375	0.224
Genotypes × Environment (GxE)	126	1448	2.519	1.7E-11***
Residuals	336	575		

***Significant at 0.001 probability levels by F test.

**Figure 1:**
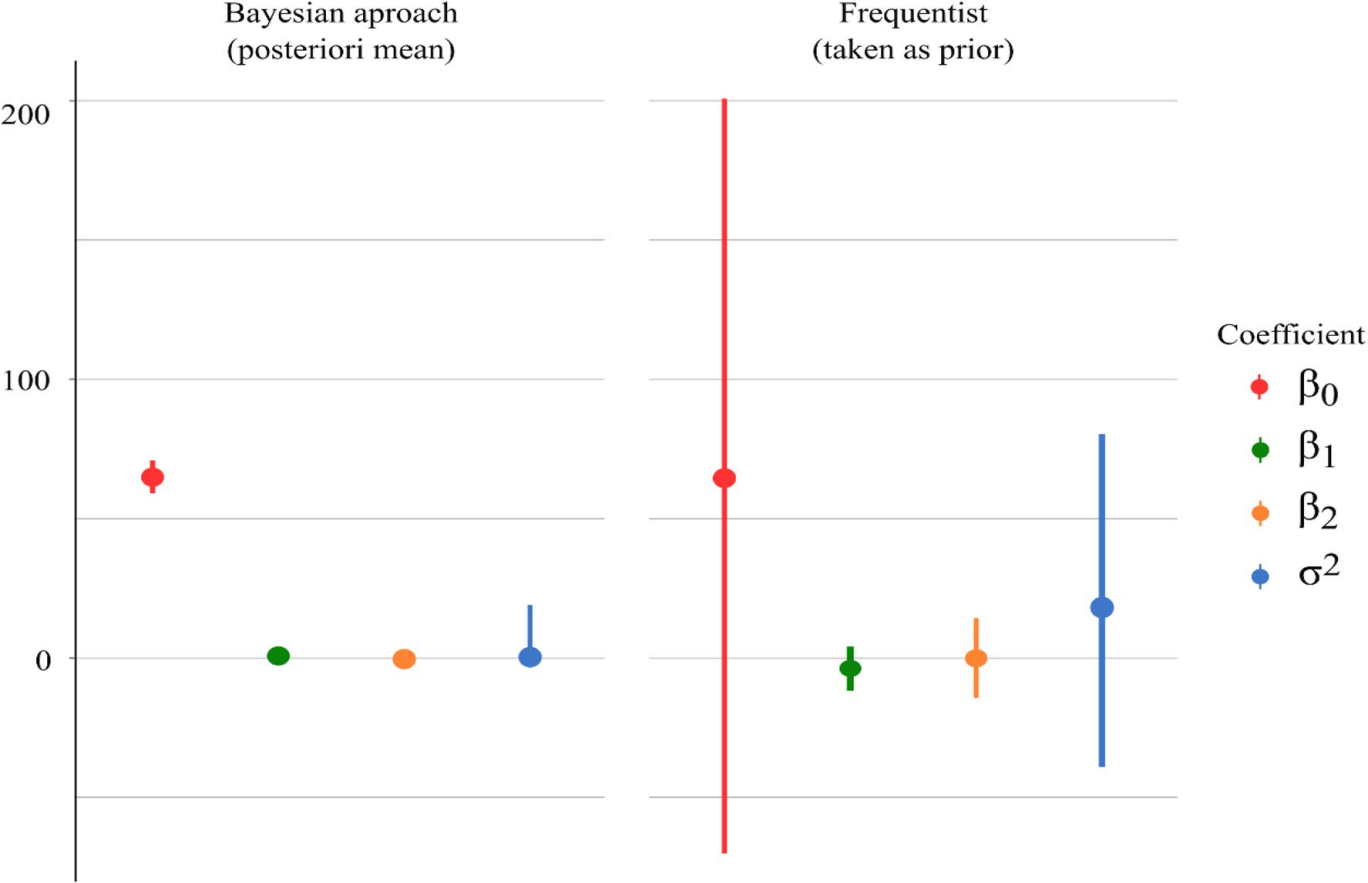
Difference between coefficient estimates, credibility intervals (Bayesian approach, left), and confidence intervals (frequentist approach, right). Coefficients estimated by the Bayesian approach were obtained after fit with the function rstanarm::stam_glm using priors from frequentist, and by the frequentist approach using the function stats::glm on previous experiments.

The causes of the GxE interaction can be attributed to physiological, and adaptive factors, related to the measurement scale of variables, among others. Which set of mechanisms allows a genotype to respond better to an environment is a question still at the frontier of science, usually trying to be answered involving molecular techniques, where the Bayesian approach is also gaining prominence. Here, we use this approach just to identify these genotypes and their response with less bias and variance. The breeder, in addition to identifying an approach that can bring better results to his pipeline, needs to adjust his model to the data. In this work, as described in the methodology, we opted a priori for frequentist approaches (Fig. 1) to obtain, with the Bayesian approach, estimates with shorter credibility intervals.

Note that in this example of any genotype used to illustrate (Fig. 1), not only were the credibility intervals much smaller compared to the confidence intervals but the estimates of some parameters were changed/corrected. This reduction in the limits of the credibility interval very well described when it was proposed^51,52^ has also been evidenced by other studies using this approach with similar models for Stability and Adaptability^28,41,43,44,53^, but not for coffee cultivation. For example, this genotype would have its negative estimate for the parameter referring to the unfavorable environment, whereas in the Bayesian approach, its estimate was close to zero (probably positive) and the error estimate much lower (parameter related to stability). This in itself is an interesting result, but it is particularly useful when the breeder is dealing with dynamic environments, where the variance of genotypes can generate wide confidence intervals in the frequentist approach, as highlighted in^54^. This was just an illustration that we believe will help breeders when choosing their models. The results specific to the genotypes studied here will be discussed throughout the text below.

**Figure 2:**
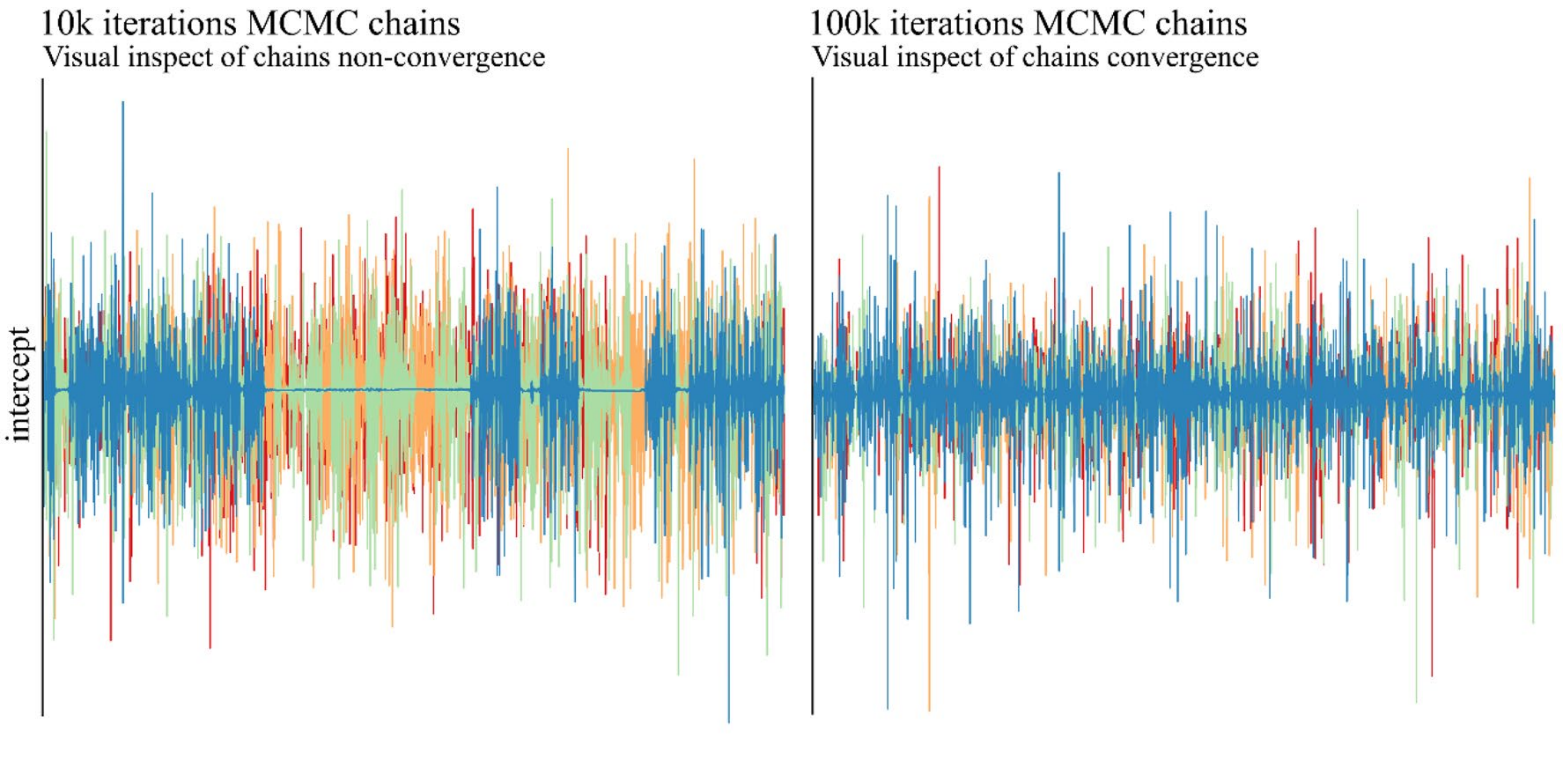
Visual inspection of chain convergence in four independent chains to take the posterior mean of each coefficient in a Bayesian Bi-segmented Regression fitted model. The left intercept coefficient from the model shows problems in convergence using only 10 thousand iterations. On the right, the same coefficient in chains of 100 thousand iterations. In both cases, burn-in was disregarded.

We would also like to highlight the process of choosing the number of iterations of the MCMC chains. As this step may require additional time to adjust the model, due to the computational power required for so many calculations, we illustrate here an example of chains from our starting point and the final number of iterations until stability in the chains is obtained (Fig. 2).

It is possible to observe that in chains with only 10,000 iterations, at times some four chains fail to estimate for the longest time the same value for the parameter. This is a problem because, with these chains, we seek precisely the marginal distribution, and this stability could be interpreted as a point estimate, which is the opposite. This can also mean overfitting the model, limiting inferences about culture to the data used here. Better discussions on this can be found at^55,56^.

After refining the model, the objective was to access the interactions between genotypes and the environment, since significant interaction between these factors had already been detected (Table 1). The edaphoclimatic conditions of each environment (E) (Supplementary Fig. S1), which present differences in climatic effect year by year, such as rainfall and temperature, can be the main font of alterations in the environmental factor that affect genotypes responses. The extreme cases are the temperatures higher than 37 ºC, which can be tolerated by *C. canephora*, using a molecular mechanism of maintenance of photoprotection and antioxidant^57,58^ reducing grain weight and yield^59^. Mean temperatures lower than 17 ºC and higher than 31 ºC, also affect the growth of C. canephora^60,61^. These dynamic environments are a challenge because although the criteria for testing cultivation value and use in the process of launching new cultivars generally consider some environments, not all are tested^62^. This puts the farmer in a situation where he depends on an often-inaccurate recommendation for his specific region.

An alternative to mitigate the influences of the GxE interaction is to recommend/use genotypes with broad adaptability and good stability. The most productive genotypes are preferred, aggregated with the characteristics already mentioned. Some coffee genotypes have been studied for tolerance traits related to the environment^45,61,63–65^. These studies have shown that genetic variability allows the identification of more stable genotypes and that they respond well to favourable environments or even tolerate more extreme conditions.

**Figure 3:**
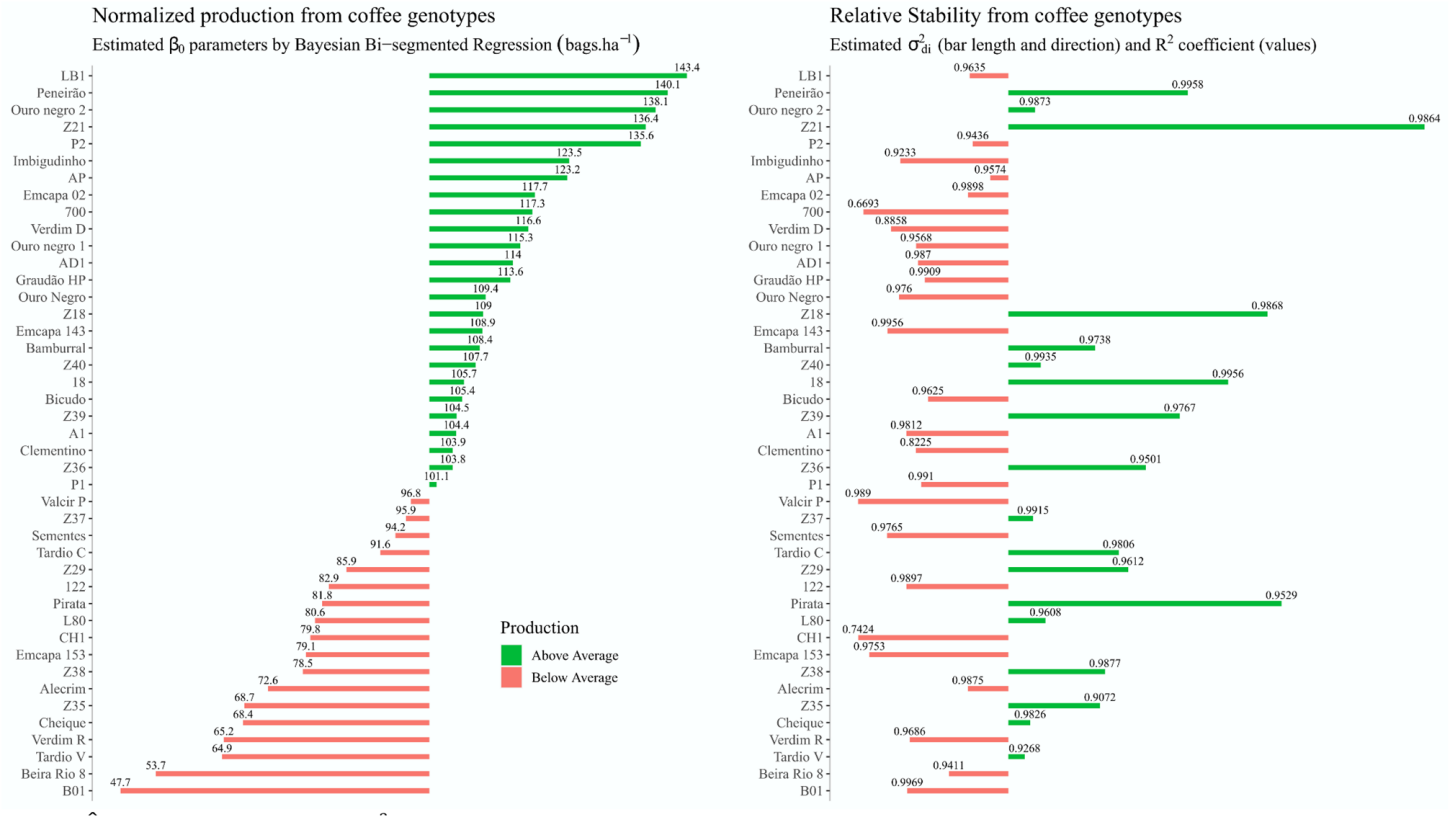
$\widehat{{{ }\beta }}_{0}$ (harvest average, at left) and $\hat{\sigma }_{di}^{2}$ (stability in the bars, with values of R^2^ of model in each bar, at right) estimates in Z-Score Normalization for C. canephora genotypes. These parameters were taken from a bisegmented Bayesian regression, modeled to describe adaptability and stability. In green, values refer to the estimate of the parameter above the average, and in red, estimates below the general average.

We gathered information about the average production of green grain bags per hectare and the $\hat{\sigma }_{di}^{2}$ parameter, together with the coefficient of determination of the model (R^2^) for *C. canephora* genotypes to highlight the most productive genotypes, which may have a genetic basis that allows more stability (Fig. 3). Where, easily a farmer could choose a genotype that would meet the desired level of production, which had good stability, with some level of confidence in the information.

It was possible to observe that the production average in the evaluated genotypes is between 96.8 and 101.1 bags^.^ha^−1^, being possible to choose several genotypes with green grain bags yield above the average. However, when observing the stability of these genotypes that have a production mean above the overall mean (Fig. 3, in the right half), it is noticed that only eight of these genotypes have good stability (represented by green bars). Thus, the genotypes Z21 (136.4 bags^.^ha-1), Peneirão (140.1 bags^.^ha-1), and Ouro negro 2 (138.1 bags^.^ha-1), ranked from the most stable to the least stable, can be chosen by farmers as the most stable and most productive (more than one standard deviation above the overall mean).

When observing the model determination coefficients (annotations above bars on the right side of Fig. 3), we found that the adjusted model has a good ability to describe our data for most genotypes. This may be an indication that the information regarding the estimates of the model parameters may have a low bias when considered by farmers for decision-making and choice of genotypes, with a majority of R^2^ higher than 0.90. Only four genotypes showed R^2^ below 0.9 (700, CH1, Verdim D, and Clementino), but since none showed good stability, nor were they among the most productive, they were not recommended in any way. Except for the Verdim D genotype, which showed good adaptability, but still was not recommended by good production, as it had low stability, with other genotypes that met the same requirements.

**Figure 4:**
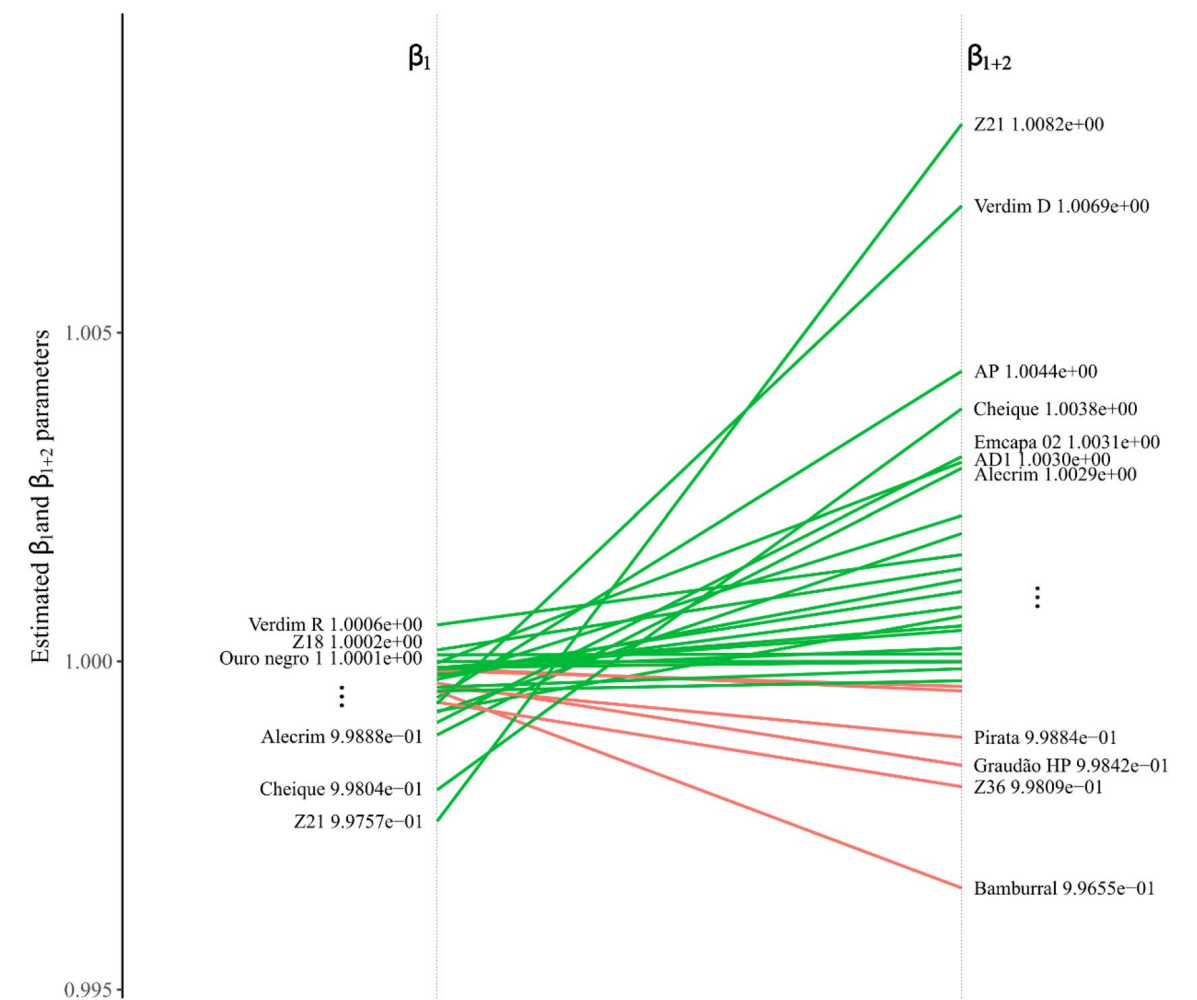
The slope between $\hat{\beta }_{i1}$ and $\hat{\beta }_{i2}$, the adaptability to the favorable environment for *C. canephora* genotypes. These parameters were taken from a bisegmented Bayesian regression, modeled to describe adaptability and stability. In green, straight refers to a positive response to a favorable environment, and in red, straight of genotypes that do not respond to the favorable environment. The slope refers to how much a genotype can respond to a favorable environment. The list of genotype names shown next to lines $\hat{\beta }_{i1}$ and $\hat{\beta }_{i2}$ is incomplete. Only names that do not overlap at the extremes were shown. The complete list of genotypes and estimated coefficient values can be found in Supplementary Table S2.

The LB1 genotype was the most productive (143.4 bags^.^ha^−1^) but showed low stability. But the farmer can still consider his adaptability, and how responsive he can be to a favorable environment. The adaptability estimates according to the model used were plotted to show the genotypes that responded positively to the favorable environment (Fig. 4).

It is possible to observe that the LB1 genotype is the twelfth among the genotypes that best respond to a favorable environment. Now, the farmer must decide whether the difference between his production and the production between the genotypes that most respond to a favorable environment compensates for his low stability. The Z21 genotype is the one that most responds to the favorable environment (Fig. 4, the straight with the highest slope in $\beta_{i2}$). However, the difference between the production of genotypes LB1 and Z21 is only ~ 5%. So, it doesn't pay to get the most productive genotype, if there is another among the top5 that responds much more to the favorable environment. However, it is necessary to note that the Z21 genotype is also the genotype whose production is severely impaired when subjected to an unfavorable environment (Fig. 4, the line with the lowest point in $\beta_{i1}$). This would be a recommended genotype for producers with a high level of technology and who will need more inputs for their environment.

Some genotypes showed low adaptability to the favorable environment (red lines on the slope between $\beta_{i1}$ and $\beta_{i2}$), and although some of these genotypes may have a production above the general average, such as Bamburral (108.4 bags^.^ha^−1^), it may not be interesting for the farmer to use this genotype, because, for example, if the precipitation is greater than expected, the genotype may not take advantage of this extra resource. On the other hand, other an average production above the general average, with stability and that do not respond as well to the genotypes also have favorable environment but can capture part of this type of resource.

Interestingly, it is possible to observe in practice a breeding program corroborating our data. For example, we do not recommend the Bamburral because it does not meet all characteristics, but this genotype has some interesting characteristics. It was used among five others to donate genetic material to compose one of the last cultivars launched, called “Tributun”, registered as number 37808 by the National Registry of Cultivars (Registro Nacional de Cultivares, RNC) by the Brazilian Ministry of Agriculture, Livestock and Food Supply (Ministério da Agricultura, Pecuária e Abastecimento, MAPA). This breeding process uses clones discovered by the farmers themselves, evaluated in the north of Espírito Santo state, Brazil^62^.

Among the stable genotypes, with good production in the unfavorable environment and which are also able to respond to improvements in the environment are cultivars 18 (105.7 bags^.^ha-1), Z18 (108.9 bags^.^ha^−1^), and mainly Ouro negro 2 (138.1 bags^.^ha^−1^) and Peneirão (140.1 bags^.^ha^−1^), which are among the most productive genotypes among those evaluated. These genotypes can serve well smaller, fewer technician farmers and also family agriculture, these farmers competitive production with the market and greater profitability.

## Conclusion

It was possible to use previous information on coffee genotypes as prior information on parameter distributions of an Adaptability and Stability model, which allowed obtaining shorter credibility intervals and good evidence of low bias in the model by the determination coefficient. After fine adjustments in the approach, it was possible to make inferences about the significant GxE interaction and to discriminate the coffee genotypes regarding production, adaptability, and stability. This is still a new approach for perennials, and since it allows more accurate estimates it can be advantageous when planning breeding programs.

The most productive and stable genotypes have an average production 2.5 times higher than the less productive genotypes and with low stability. The Z21 genotype is recommended to compose part of selected genetic material for highly technical farmers, as it responds very well to the favorable environment, being one of the most productive and with excellent stability. While still maintaining high production and stability, but with lower adaptability, the Ouro Preto 2 and Peneirão genotypes can add to the cultivated clones serving small farmers with lower technology levels, or environments that will not offer many resources for the plants.

## Supplementary Information

Supplementary Information.

## Data Availability

The full phenotypic information, breeding values, scripts, and chains generated used in this study, have been submitted to the Open Science Framework and were awarded the public DOI identifier: https://doi.org/10.17605/OSF.IO/D8T2R.
