# Supplementary material for: Adaptability and stability of Coffea canephora to dynamic environments using the Bayesian approach
Source: Sci Rep. 2022 Jul 8;12:11608. doi: 10.1038/s41598-022-15190-x (PMC9270379; doi:10.1038/s41598-022-15190-x)

**Supplementary Table 1** Identification of 43 Coffea canephora genotypes. Nova Venécia, ES - Brazil.

| Code | Name | Code | Name | Code | Name |
| --- | --- | --- | --- | --- | --- |
| 1 | Verdim R | 16 | Pirata | 30 | A1 |
| 2 | B01 | 17 | Peneirão | 31 | Cheique |
| 3 | Bicudo | 18 | Z39 | 32 | P2 |
| 4 | Alecrim | 19 | Z35 | 33 | Emcapa 02 |
| 5 | 700 | 20 | Z40 | 34 | Emcapa 153 |
| 6 | CH1 | 21 | Z29 | 35 | P1 |
| 7 | Imbigudinho | 22 | Z38 | 36 | LB1 |
| 8 | AD1 | 23 | Z18 | 37 | 122 |
| 9 | Graudão HP | 24 | Z37 | 38 | Verdim D |
| 10 | Valcir P | 25 | Z21 | 39 | Sementes |
| 11 | Beira Rio 8 | 26 | Z36 | 40 | Emcapa 143 |
| 12 | Tardio V | 27 | Ouro Negro | 41 | Ouro negro 1 |
| 13 | AP | 28 | 18 | 42 | Ouro negro 2 |
| 14 | L80 | 29 | Tardio C | 43 | Clementino |
| 15 | Bamburral |  |  |  |  |

Genotype 33 belongs to cv. Emcapa 8111 and genotypes 34 and 39 to cv. Emcapa 8131 (Bragança et al., 2001). Genotypes 1, 11, 15, 16, 30 and 43 belong to cv. Tributun (Partelli et al., 2020) and 30 and 35 to cv. Andina (Partelli et al., 2019).

**Supplementary Table 2.** Complete list of genotypes and coefficient estimates, along with lower and upper confidence intervals.

| **Genotypes** | **L_Beta_0** | **Beta_0** | **U_Beta_0** | **L_Beta_1** | **Beta_1** | **U_Beta_1** | **L_1+2** | **B_1+2** | **U_1+2** | **L_Sigma** | **Sigma** | **U_Sigma** | **R^2** |
| --- | --- | --- | --- | --- | --- | --- | --- | --- | --- | --- | --- | --- | --- |
| Verdim R | 51,90 | 65,18 | 77,48 | 0,4533 | 1,0006 | 1,4535 | -1,796 | 0,001623699 | 1,620 | 0,0768 | 1,0443 | 30,6165 | 0,9686 |
| Z18 | 89,65 | 108,99 | 127,39 | 0,4988 | 1,0002 | 1,4084 | -3,306 | 0,001408779 | 3,657 | 0,1367 | 8,2940 | 55,6061 | 0,9868 |
| Ouro negro 1 | 103,90 | 115,26 | 126,38 | 0,5940 | 1,0001 | 1,3372 | -0,697 | 0,000114772 | 0,749 | 0,0407 | 1,1767 | 41,2504 | 0,9568 |
| Z39 | 89,47 | 104,48 | 118,70 | 0,5829 | 1,0000 | 1,3420 | -1,236 | -2,50E-08 | 1,427 | 0,0000 | 6,5203 | 51,0665 | 0,9767 |
| Sementes | 85,34 | 94,18 | 102,96 | 0,6771 | 1,0000 | 1,2753 | -0,758 | 5,20E-10 | 0,766 | 0,0000 | 0,5869 | 33,4458 | 0,9765 |
| Valcir P | 95,16 | 96,79 | 98,33 | 0,9276 | 1,0000 | 1,0651 | -0,107 | -1,01E-11 | 0,098 | 0,0000 | 0,0000 | 9,8876 | 0,9890 |
| Ouro Negro | 108,29 | 109,39 | 110,56 | 0,9556 | 1,0000 | 1,0406 | -0,099 | -7,93E-11 | 0,100 | 0,0000 | 0,8321 | 9,4909 | 0,9760 |
| Emcapa 143 | 99,13 | 108,85 | 118,04 | 0,6532 | 1,0000 | 1,2922 | -1,413 | 3,95E-11 | 1,354 | 0,0000 | 0,5950 | 32,2972 | 0,9956 |
| Z38 | 68,19 | 78,54 | 88,62 | 0,6424 | 1,0000 | 1,3048 | -1,054 | 4,42E-09 | 1,100 | 0,0000 | 5,0030 | 37,0881 | 0,9877 |
| CH1 | 77,07 | 79,80 | 82,59 | 0,9113 | 1,0000 | 1,0810 | -0,262 | 1,97E-09 | 0,257 | 0,0000 | 0,0014 | 17,3599 | 0,7424 |
| Z37 | 88,69 | 95,93 | 103,24 | 0,7504 | 1,0000 | 1,2129 | -0,715 | 4,65E-12 | 0,718 | 0,0000 | 3,5466 | 31,3136 | 0,9915 |
| Z35 | 58,91 | 68,68 | 78,68 | 0,6507 | 1,0000 | 1,2966 | -0,581 | -6,62E-09 | 0,612 | 0,0000 | 4,9022 | 38,8307 | 0,9072 |
| Emcapa 153 | 71,43 | 79,06 | 86,63 | 0,7436 | 1,0000 | 1,2081 | -1,623 | -7,87E-09 | 1,629 | 0,0000 | 0,2288 | 34,0741 | 0,9753 |
| 122 | 70,25 | 82,92 | 95,70 | 0,6090 | 1,0000 | 1,3295 | -1,190 | 5,09E-09 | 1,311 | 0,0000 | 0,9813 | 47,5404 | 0,9897 |
| B01 | 70,37 | 81,51 | 86,43 | 0,6466 | 1,0000 | 1,2947 | -0,906 | 5,14E-10 | 0,817 | 0,0000 | 2,9845 | 31,8765 | 0,9775 |
| L80 | 71,62 | 80,59 | 89,25 | 0,6536 | 1,0000 | 1,2836 | -0,904 | 5,17E-10 | 0,826 | 0,0000 | 3,7963 | 29,5151 | 0,9608 |
| Z29 | 75,26 | 85,90 | 96,63 | 0,6376 | 1,0000 | 1,2997 | -1,043 | 6,42E-09 | 1,111 | 0,0000 | 5,4723 | 41,2446 | 0,9612 |
| 700 | 105,59 | 117,28 | 129,94 | 0,5684 | 1,0000 | 1,2967 | -0,866 | -9,98E-06 | 0,951 | 0,0010 | 0,1085 | 44,1100 | 0,6693 |
| AD1 | 98,03 | 113,99 | 128,78 | 0,5201 | 1,0000 | 1,4117 | -109,964 | 0,003028782 | 121,192 | 0,0097 | 1,2156 | 41,4373 | 0,9870 |
| Beira Rio 8 | 40,45 | 53,71 | 66,99 | 0,5486 | 0,9999 | 1,3877 | -0,752 | -3,49E-06 | 0,809 | 0,0377 | 1,8399 | 47,1954 | 0,9411 |
| Tardio C | 80,81 | 91,62 | 102,41 | 0,5864 | 0,9999 | 1,3527 | -0,752 | 0,000541701 | 0,760 | 0,0431 | 5,2799 | 36,2016 | 0,9806 |
| Bicudo | 93,09 | 105,44 | 116,76 | 0,5545 | 0,9999 | 1,3631 | -1,887 | -0,00044882 | 1,738 | 0,0296 | 1,4184 | 35,1941 | 0,9625 |
| 18 | 91,32 | 105,73 | 120,01 | 0,5363 | 0,9999 | 1,3815 | -2,186 | 0,000472138 | 2,317 | 0,0838 | 7,4978 | 50,0855 | 0,9956 |
| P2 | 118,98 | 135,61 | 151,58 | 0,5125 | 0,9999 | 1,3977 | -0,917 | 0,000203124 | 0,993 | 0,0224 | 2,3252 | 56,1217 | 0,9436 |
| Z40 | 85,00 | 107,69 | 125,78 | 0,1033 | 0,9999 | 1,6835 | -5,312 | -0,000381413 | 4,188 | 0,0417 | 3,7005 | 25,7105 | 0,9935 |
| Imbigudinho | 111,04 | 123,51 | 135,91 | 0,6596 | 0,9998 | 1,2792 | -1,087 | 0,001062839 | 1,164 | 0,0339 | 0,8563 | 48,5682 | 0,9233 |
| Ouro negro 2 | 103,88 | 138,12 | 169,27 | 0,2956 | 0,9998 | 1,5727 | -2,053 | 0,000826369 | 2,422 | 0,1637 | 3,5872 | 77,2535 | 0,9873 |
| LB1 | 120,70 | 143,38 | 164,20 | 0,3916 | 0,9997 | 1,4899 | -2,925 | 0,001241751 | 3,359 | 0,1893 | 2,2617 | 57,4460 | 0,9635 |
| P1 | 92,25 | 101,13 | 109,69 | 0,6519 | 0,9997 | 1,2965 | -1,046 | 0,002217022 | 1,007 | 0,0025 | 1,2784 | 29,7986 | 0,9910 |
| Graudão HP | 97,37 | 113,58 | 128,16 | 0,3018 | 0,9997 | 1,5695 | -2,104 | -0,001582651 | 1,856 | 0,1096 | 1,3534 | 28,6589 | 0,9909 |
| Pirata | 65,07 | 81,81 | 98,45 | 0,5317 | 0,9996 | 1,3930 | -1,375 | -0,001156055 | 1,488 | 0,2202 | 8,5795 | 56,5817 | 0,9529 |
| Clementino | 92,62 | 103,86 | 114,51 | 0,4954 | 0,9996 | 1,4074 | -1,296 | -0,000111931 | 1,184 | 0,0927 | 1,1699 | 30,0454 | 0,8225 |
| A1 | 89,05 | 104,44 | 117,43 | 0,2953 | 0,9995 | 1,5392 | -1,982 | -0,00029487 | 1,593 | 0,0592 | 0,9766 | 25,9829 | 0,9812 |
| Bamburral | 86,57 | 108,41 | 126,24 | 0,2058 | 0,9995 | 1,6022 | -6,557 | -0,003451485 | 5,279 | 0,0630 | 4,8016 | 32,4962 | 0,9738 |
| AP | 96,38 | 123,19 | 146,03 | 0,2034 | 0,9994 | 1,6117 | -14,684 | 0,004414576 | 17,800 | 0,2921 | 2,6777 | 46,2788 | 0,9574 |
| Z36 | 90,42 | 103,84 | 116,55 | 0,4956 | 0,9994 | 1,4179 | -2,305 | -0,001908797 | 2,182 | 0,1267 | 5,8324 | 37,1184 | 0,9501 |
| Verdim D | 102,13 | 116,59 | 129,02 | 0,4478 | 0,9994 | 1,3997 | -3,983 | 0,006932884 | 3,154 | 0,0308 | 0,6682 | 32,4196 | 0,8858 |
| Tardio V | 44,29 | 64,88 | 85,67 | 0,3884 | 0,9992 | 1,4709 | -1,020 | 0,000688203 | 1,137 | 0,1968 | 3,3732 | 68,6705 | 0,9268 |
| Peneirão | 106,77 | 140,13 | 165,28 | -0,1680 | 0,9992 | 1,8444 | -1,993 | 0,00194729 | 2,738 | 0,1084 | 6,6818 | 43,9044 | 0,9958 |
| Emcapa 02 | 89,97 | 117,71 | 142,78 | 0,3640 | 0,9991 | 1,5023 | -2,119 | 0,0031138 | 2,544 | 0,0998 | 2,2279 | 61,6495 | 0,9898 |
| Alecrim | 45,02 | 72,65 | 94,41 | -0,0005 | 0,9989 | 1,7125 | -1,919 | 0,002937727 | 2,596 | 0,1599 | 2,2245 | 46,0901 | 0,9875 |
| Cheique | 34,08 | 68,45 | 96,63 | -0,1077 | 0,9980 | 1,8185 | -2,057 | 0,003843218 | 2,659 | 0,3007 | 3,4871 | 57,9318 | 0,9826 |
| Z21 | 72,73 | 136,43 | 186,20 | -0,0499 | 0,9976 | 1,7877 | -3,516 | 0,008178074 | 4,548 | 0,3579 | 11,4798 | 70,2541 | 0,9864 |

**Supplementary Figure 1** Average, maximum and minimum meteorological data referring to temperature, radiation, relative humidity and precipitation at the site of the experiments, during the evaluation period of coffee genotypes *C. canephora* from January 2014 to June 2019, for modeling adaptability and stability.


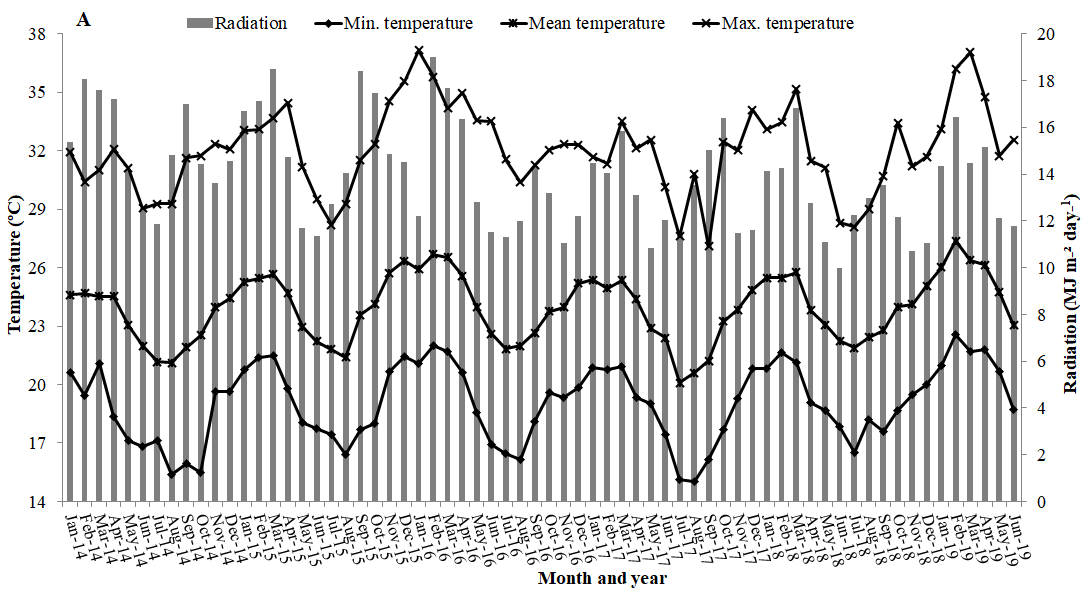

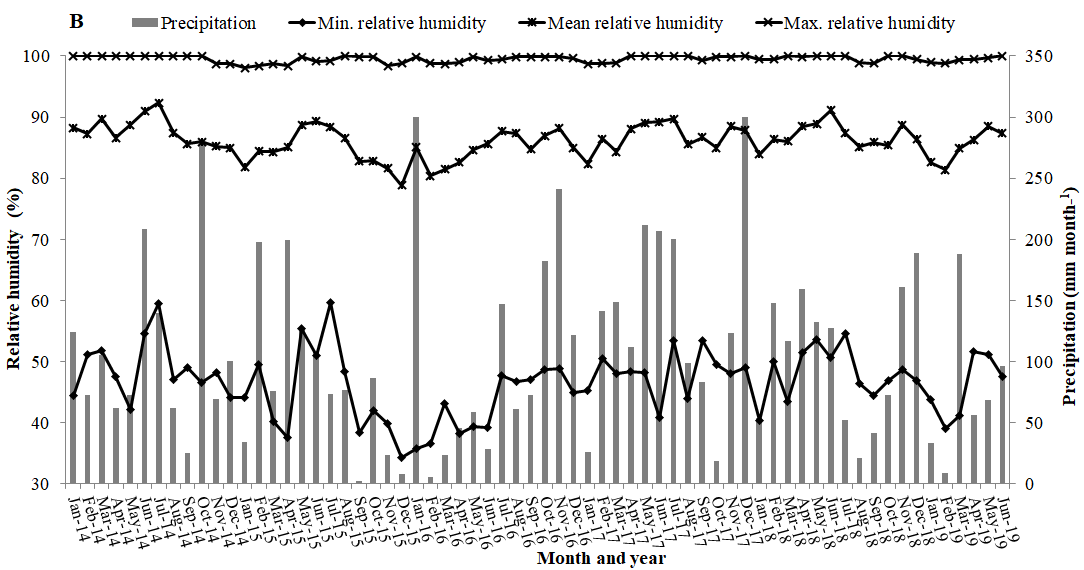

Supplement: Supplementary file 1 — Supplementary Information. [file 41598_2022_15190_MOESM1_ESM.docx]
